# Psychosocial determinants of sexual norms and their impact on sexual debut in Polish adolescents

**DOI:** 10.1007/s00038-020-01470-8

**Published:** 2020-09-02

**Authors:** Zbigniew Izdebski, Krzysztof Wąż, Anna Kowalewska, Joanna Mazur

**Affiliations:** 1 Department of Biomedical Foundations of Development and Sexology, Faculty of Education, University of Warsaw, Warsaw, Poland; 2 Institute of Pedagogy, University of Zielona Góra, Zielona Góra, Poland; 3 Department of Humanization in Medicine and Sexology, Institute of Health Science, University of Zielona Góra, Zielona Góra, Poland

**Keywords:** Adolescents, Sexual initiation, Moral norms, Gender norms, Psychosocial determinants

## Abstract

**Objectives:**

In an attempt to describe composite scales relating to sexual norms, we present their structure, psychosocial determinants, and the association with adolescent sexual initiation.

**Methods:**

A representative sample of Polish students was surveyed in 2015 (*n* = 1024, mean age 17.8 ± 0.31). Three scales of sexual norms were identified using principal component analysis. Logistic regression and path models were applied.

**Results:**

The scales concerning stereotype gender norms, as well as restrictive and permissive sexual norms, were developed. Restrictive norms lead to later sexual initiation and were more frequent in combination with love for the first partner. The variability of all the indices was significantly correlated with the peer environment. A number of direct and indirect paths were found among the above indices, family relationships (support, communication), and socio-emotional strengths, such as the meaning of life, coherence, and self-esteem.

**Conclusions:**

The norms of sexual morality should be included in the analysis of the determinants of adolescent sexual behaviours along with an investigation of complex causal models. The results may contribute to improvement in sexual health promotion.

**Electronic supplementary material:**

The online version of this article (10.1007/s00038-020-01470-8) contains supplementary material, which is available to authorized users.

## Introduction

Adolescence is a period of intensive changes related to the transition from the period of childhood to adulthood. The process is accompanied by biological, psychological, social, spiritual, and sexual changes. Puberty occurs earlier than emotional and social maturity (Jaczewski 2015; Savolainen et al. 2015). Undertaking different forms of sexual expression is a form of socializing sexuality and has an influence on the course of development in this period of life. The process of acquiring sexual experience includes pre-initiation experiences, entering into romantic relationships and dating, as well as sexual initiation (De Graaf et al. 2011; Collins et al. 2009). Early sexual initiation can pose a threat to adolescent development and health, including sexual health. It also increases the risk associated with engaging in risky behaviours on the part of adolescents (Moreau 2019a, b; Savolainen et al. 2015). The integration of the physical, emotional, intellectual, and social dimensions of sexual health and their positive impact on personality development, communication, and love is underscored in WHO documents (1975). Sexual initiation is determined by many factors including biological, psychosocial, and cultural ones.

The focus of research on sexual and reproductive health in the last few years has been on their links with social norms (Harvey 2013; Pulerwitz et al. 2019). The issue of social norms is very complex and defined in various ways. According to Zimbardo and Gerrig (1996), a social norm means the expectations of a group regarding acceptable and appropriate attitudes and behaviours on the parts of its members. In some papers, the distinction between restrictive and permissive norms, or messages related to sexuality, has been introduced. Restrictive norms are associated with the concept of sin or something disturbing, a risk that one should be cautious of. Conversely, permissive norms are described as relating to an opportunity to gain new experiences (Boratav and Cavdar 2012). The theoretical grounds for research with regard to norms stem from the ecological model of human development (Bronfenbrenner 1994). Social learning theory (Bandura 1998) and reasoned action theory (Fishbein and Ajzen 1975; Azjen 1991) have also been applied. Pulerwitz et al. (2019) developed the social norms theory to be used as a foundation for devising preventive measures in the area of sexual and procreative health.

Norms enable regulating functioning in society and can change in terms of its transformation (Muniruzzaman 2017). Christianity has played an important role in shaping attitudes towards sex in western culture. Starting in the 1960s, restrictive approaches with regard to sex gave away to more permissive attitudes (Giddens 1992). Rapid changes in the sexual moral norms of adolescents, particularly girls, have been observed over the last few years in many countries including Poland (Zurbriggen et al. 2007). In the era of the growing importance of the Internet, “media sexualization” has occurred (Rideout et al. 2010; Merskin 2014) as is visible in the “culture of exposure” (what used to be private is now public), the “democratization of desire” (widespread availability of means to express sexuality), and “porn chic” (the pervasion of pornography into representations of popular culture) (Giddens 1992).

In addition, many countries, especially those of Eastern and Central Europe, lack a climate conducive for popularizing sex education that is adjusted to the child and adolescent developmental stages. The results presented in a report by the International Planned Parenthood Federation European Network (IPPF EN) and the German Federal Centre for Health Education (Ketting and Ivanova 2018) show a noticeable opposition to sex education. Poland also has a model of school sex education which focuses on abstinence, and bills are submitted to parliament every few years with the intention of intimidating sex educators and teachers, and to ban abortion completely. These measures result from a fear of accelerating sexual initiation. This fear is repudiated by data from the Netherlands and Switzerland, where initiation indicators have been the lowest among the countries analysed, despite a very extensive multiannual sex education programme (Ketting and Ivanova 2018).

It seems that understanding the determinants of sexual initiation requires an improved knowledge of not only social, but also cultural, factors. As far as the latter is concerned, it is worth considering their indirect and direct psychosocial determinants and measurement methods. The measurement tools should be adjusted to the continuously changing world and the specificity resulting from the age of the respondents, as well as their countries of origin.

The study aimed to examine the associations between sexual norms, selected psychosocial factors, and the sexual initiation of older adolescents. Based on the previous analyses of the single items measuring social norms related to sexuality (Wąż 2019), the following detailed research topics were decided upon:

1. To establish whether or not any scales with good psychometric qualities can be extracted from statements on the social and moral norms related to sexuality.
2. To investigate the association of those scales with selected indicators of sexual initiation.
3. To build a causal model based on the identified scales relating to sexual norms, the selected characteristics of the family and peer environment, and the individual assets defined as socio-emotional strengths.

## Methods

### Participants

The results of the survey conducted as part of the “Adolescents and Health” project were further analysed (Izdebski et al. 2017). A questionnaire containing the authors’ questions, along with questions used in the Health Behaviour in School-aged Children (HBSC) study following the 2013/2014 protocol (Currie et al. 2014; Mazur and Malkowska-Szkutnik (eds) 2018), was used.

The Adolescents and Health survey was carried out in 2015 among second grade secondary school students. The answers relating to all statements on sexual norms were derived from 1024 students (53.6% boys and 43.6% girls), meeting the age criterion of 16.5 to 18.5 years (mean 17.8 ± 0.31).

The survey study respected the principle of anonymity. The respondents had been informed about the purpose of the study and could refuse to take part without stating a reason.

### Main outcomes and instruments

*Pre*-*initiation sexual* experiences were defined as experiences preceding the first sexual intercourse, such as passionate kissing and petting. The respondents indicated whether they had previously had such experiences and stated their age at the time of any such occurrence.

The *sexual initiation* part contained two questions. The respondents were asked if they had already had sexual intercourse (yes, no) and, if so, at what age.

The respondents who had already experienced sexual initiation responded on a 4-point scale whether it was accompanied by a feeling of love for their first sexual intercourse partner (ranging from *definitely yes* to *definitely no*).

Three separate binary variables (1/0) were defined, where value 1 meant: being sexually initiated, early initiation defined as before the age of 15, and initiation accompanied definitely by the feeling of love for the partner. To describe our sample, a division was also made into three groups of responders: those who had not had any sexual experiences, those who had only pre-initiation experiences, and those after the first experience of sexual intercourse.

### Items on sexual moral norms

In the original version of the questionnaire, there were two blocks of questions, each containing eight statements with regard to intimate contact. The first block concerned stereotypical views on gender roles, while the second related to the permissibility of various behaviours. The respondents indicated how much they agreed with the following statements on a five-point scale. Labels appeared only with extreme answers: *strongly disagree* and *strongly agree*, which eliminated labelling the middle category as a neutral one. A detailed description of those scales is included in the part relating to the survey results relating to the specified research topics. The wording of the items can be found in the supplementary electronic part (Table S1).

### Psychosocial determinants

The following scales relating to strengths and social relationships and tested previously in Poland were taken into account as potential psychosocial determinants:

- sense of the meaning of life according to the Polish 4-item Purposes in Life scale based on the Schulenberg approach (2016)—range 0–24; alpha = 0.809; mean 17.11 ± 4.61.
- sense of self-esteem on the 10-point Rosenberg (1965) scale—range 0–30; alpha = 0.845; mean 18.83 ± 5.18.
- sense of coherence according to the Polish 11-item Sense of Coherence (SOC) scale developed by Zwoliński (1999)—range 0–30; alpha = 0.856; mean 21.38 ± 7.62.
- family communication quality according to a 4-item scale sourced from the Family Dynamics Measure II scale (Lasky et al. 1985)—range 0–16; alpha = 0.832; mean 11.03 ± 3.28
- family support according to a 4-item scale sourced from Multidimensional Scale of Perceived Social Support (Zimet and Grodaon 1988)—range 0–24; alpha = 0.914; mean 17.04 ± 5.85.
- peer support according to a 4-item scale taken from the same source as above—range 0–24; alpha = 0.913; mean 16.88 ± 5.31.

### Statistical analysis

**Fig. 1:**
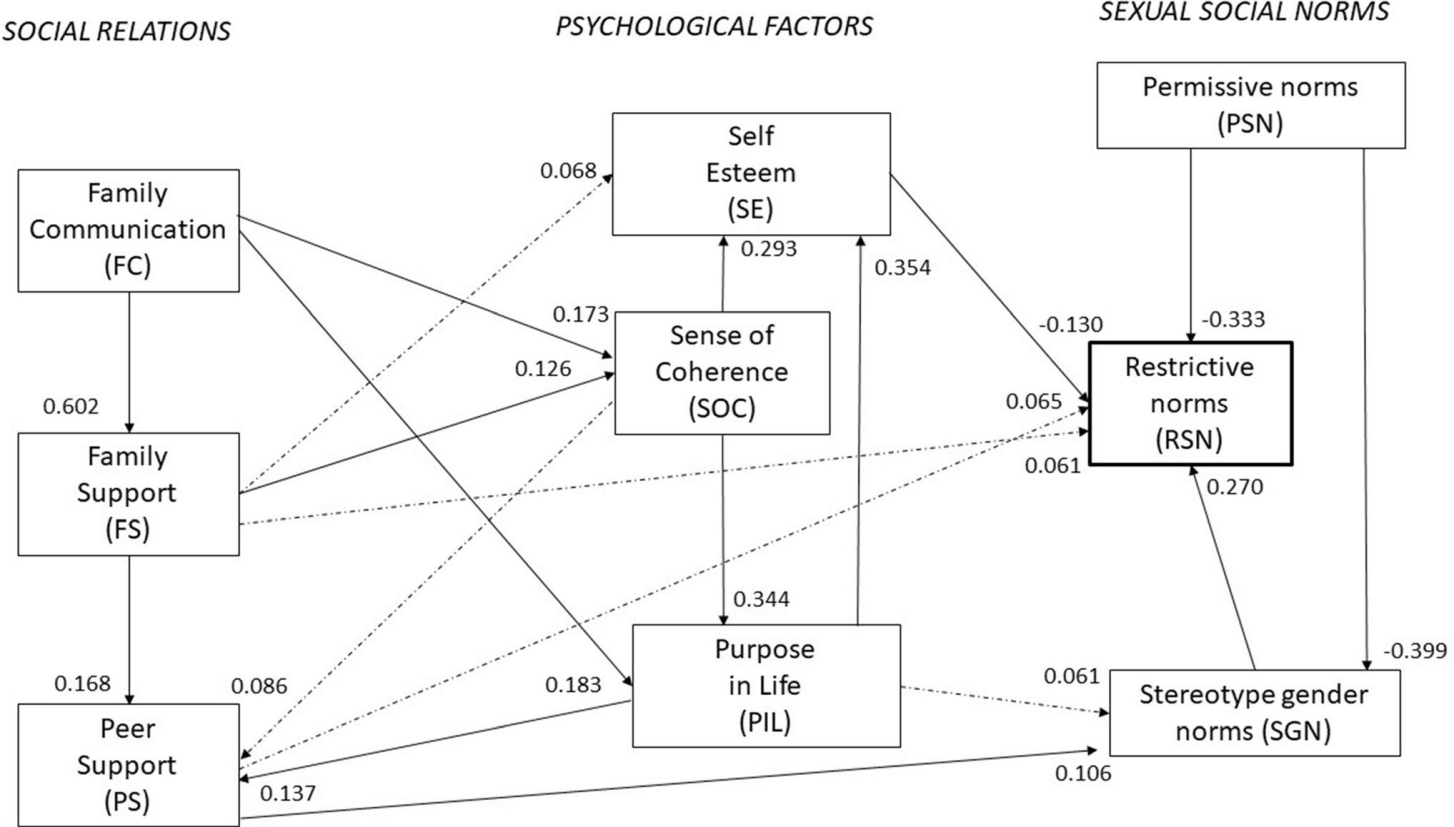
Path model for the sexual norms (Poland 2015). Arrows represent the standardized regression weights significant in the total sample: solid lines at *p * < 0.01 and dotted lines at 0.01 = <*p *< 0.05

In examining the psychometric properties of the scales, their structure was assessed using the PCA—principal component analysis with varimax rotation—and their internal consistency was evaluated using Cronbach’s alpha. The correlations among the scales were evaluated using Spearman’s correlation coefficient. Between-group differences for continuous variables were assessed using the one-way ANOVA or nonparametric tests, while, for categorical variables, the chi-squared test was applied. The association among three indicators of sexual initiation and the indices of sexual norms was examined using logistic regression adjusted for gender, providing the odds ratio (OR) with a 95% confidence interval (CI). Structural equation modelling (SEM) was applied, and a simple path model was estimated according to the diagram shown in Fig. 1. The direction of the relationship was pre-assumed and verified in terms of the model with the help of modification indices. The maximum likelihood method was applied, along with the following model goodness-of-fit statistics: the TLI (Tucker–Lewis index), the CFI (comparative fit index), and the RMSEA (root-mean-square error of approximation). The assumption was that in a good model RMSEA should not exceed a value of 0.05. The IBM SPSS software with AMOS was employed.

## Results

### Identifying scales relating to sexual norms

The analyses conducted allowed for identifying three scales. Five questions from the initial first block (and one question from the second) were rejected. Detailed psychometric properties of the identified scales, along with the mean values in the examined sample, are shown in Table 1. All the scales had a single-factor structure, with the restrictive sexual scale being the most reliable one.

**Table 1:** Properties of the scales related to sexual norms (*N *= 1024) (Poland 2015)

Name of scale	Items/range	Cronbach’s alpha	Explained variance* (%)	Mean ± SD			p**
				Total	Boys	Girls	
Stereotype gender norms	3 (0–12)	0.688	61.70	8.66 ± 2.96	7.98 ± 3.10	9.46 ± 2.58	< 0.001
Restrictive sexual norms	4 (0–16)	0.720	55.29	12.33 ± 3.77	11.11 ± 3.91	13.73 ± 3.05	< 0.001
Permissive sexual norms	3 (0–12)	0.562	53.76	2.88 ± 2.64	3.61 ± 2.80	2.03 ± 2.14	< 0.001

*Principal components analysis, one factor

**Mann–Whitney’s test for gender comparison

The first scale, consisting of four statements, was entitled “*stereotypical gender norms*”. The high value of the scale reflected the stereotypical perception of those roles based on the patriarchal model. The second scale, composed of three items, entitled “restrictive sexual norms”, included statements supporting romantic relationships and moral disapproval of premarital sex. The third scale, comprising three statements, entitled “permissive sexual norms”, concerns the use of media in intimate relationships.

### Sexual initiation and its correlation with moral norms

In the examined group of older adolescents, 64.6% had already had their first sexual experiences—one in four had (25.7%) only had pre-initiation experiences, while 38.9% had gone through full sexual initiation (Table 2).

**Table 2:** Indicators of sexual debut (Poland, 2015)

	Total	Boys	Girls	p*
*Sexual experiences (N* = *1017)*—*%*				
None	35.4	33.3	37.8	0.107
Pre-initiation	25.7	24.8	26.6	
Sexual initiation	38.9	41.9	35.6	
*Being in love with the first sexual partner (N*-*405)*—*%*				
Definitely no	8.9	12.8	3.5	< 0.001
Rather no	12.6	19.1	3.5	
Rather yes	18.0	20.4	14.8	
Definitely yes	60.5	47.7	78.2	
*Age of first intercourse (N* = *405)*				
≤ 14 years	15.7	19.5	10.5	0.002
15–16 years	42.0	44.9	38.0	
17–18 years	42.3	35.6	51.5	

*Chi-square test

Despite the differences in the respective percentages, a sample of this size did not show gender-related differences when comparing three groups of adolescents (chi-sq = 4.477; df = 2; *p *= 0.107). Girls declared a feeling of love for their first sexual partner more frequently than did boys (chi-sq = 46.267; df = 3; *p *< 0.001). The mean sexual initiation age was 15.87 ± 1.62. Girls experienced sexual initiation about 8 months later than boys (*p *< 0.001 in a nonparametric test).

An analysis of the association among selected indicators of sexual initiation and the perception of sexual moral norms (adjusted for gender) indicates the strongest correlation with restrictive sexual norms (Table 3).

**Table 3:** The impact of sexual norms on sexual initiation (Poland 2015)

	Model—dependent variable					
	Having sexual intercourse		Sexual intercourse before age of 15		Definitely in love with the first sexual partner	
	OR 95% CI	*p*	OR 95% CI	*p*	OR 95% CI	*p*
Stereotype gender norms	1.008 (0.965–1.053)	0.723	0.939 (0.864–1.021)	0.143	1.124 (1.047–1.207)	0.001
Restrictive sexual norms	0.941 (0.908–0.975)	0.001	0.876 (0.820–0.935)	< 0.001	1.246 (1.168–1.328)	< 0.001
Permissive sexual norms	1.011 (0.962–1.062)	0.671	1.017 (0.924–1.119)	0.734	0.846 (0.783–0.917)	< 0.001

*OR* odds ratio estimated in logistic regression adjusted for gender, *CI* confidence interval

Those who achieved a high score on the restrictive sexual norms scale started their sexual life later. They also waited for a partner with whom they would be in love. The high results on the permissive sexual norms scale were related to less frequent initiation with love, while in case of stereotypical gender norms scale with more frequent.

### Sexual norms and selected psychosocial factors

The three sexual morality scales are significantly correlated with each other. Restrictive sexual norms and stereotypical gender norms are positively correlated with each other (rho = 0.398), and permissive sexual norms are negatively correlated with the two above scales (− 0.435 and − 0.347, respectively)—Table 4.

**Table 4:** Correlation between scales of sexual norms and psychosocial scales (Poland 2015)

		Stereotype gender norms	Restrictive sexual norms	Permissive sexual norms
Quality of family communication	rho	0.070	0.049	− 0.065
	(*p*)	(0.025)	(0.116)	(0.038)
Family support	rho	0.090	0.070	− 0.096
	(*p*)	(0.004)	(0.025)	(0.002)
Peer support	rho	0.111	0.094	− 0.054
	(*p*)	(0.000)	(0.003)	(0.081)
Sense of coherence	rho	0.014	− 0.017	− 0.027
	(*p*)	(0.655)	(0.593)	(0.394)
Purpose in life	rho	0.101	0.041	− 0.031
	(*p*)	(0.001)	(0.189)	(0.323)
Self-esteem	rho	− 0.014	− 0.104	0.014
	(*p*)	(0.651)	(0.001)	(0.662)

Rho—Spearman’s correlation; *p*—two-side significance level

Looking at the potential determinants of all three moral norms indices, the highest correlation is found with the peer environment. Permissive sexual norms show a significant weak correlation with social, but not individual, factors. The correlation between the purpose in life and stereotypical gender norms (rho = 0.101) as well as a negative correlation between the sense of self-esteem and restrictive norms (rho = − 0.104) is also relatively higher.

A path model, shown in Fig. 1, was obtained when looking for the direct and indirect associations among the nine analysed scales (Table S2).

We looked for the sources of the variability in restrictive sexual norms which most strongly correlated with sexual initiation. Nineteen paths indicated statistically significant correlations. The model comprises seven linear equations. Permissive norms affect the variability of the restrictive sexual scale and stereotype gender norms with relatively high path coefficients, but still rather low in absolute value. Stereotypical gender norms are also strong predictors (positive) of restrictive sexual norms, as well as self-esteem (negative). The peer environment remained a statistically significant (weak) predictor and seemed to be influenced by the sense of coherence, the sense of the purpose in life, and family support. This means that social and individual factors may indirectly shape moral norms (Fig. 1). The estimated path model has satisfactory goodness-of-fit parameters. The TLI is 0.988 and the CFI is 0.994, while the RMSEA equals 0.024.

### Gender-dependent differences

The analysis of gender-related differences was outside the scope of the main research topics. However, it should be noted that girls showed significantly higher scores on the indices of restrictive and permissive norms. The index of stereotypical norms was higher in boys, and so were the scores of five of the psychosocial indices. No difference was found between boys and girls only in the case of family support. Gender-specific path models for both genders (according to Fig. 1 and the RMSEA) had even better fit parameters (0.022 for boys and 0.017 for girls), even though some correlation paths sporadically ceased to be important. For example, the peer environment is the significant determinant of the variability of the gender norms scale only in girls, while purpose in life is so only in boys (Table S2).

## Discussion

### Summary of main findings

This paper is a continuation of our research on the norms and beliefs of adolescents regarding sexuality. Whereas in our previous research we focused on analysing particular statements, we have now attempted to identify complex constructs describing norms and beliefs regarding sexuality. The dispute between more and less restrictive sexual norms was at heart of our interest. As emphasized in the Introduction, the process of the socialization of contemporary youth depends on a string of cultural factors, and contemporary media may play an increasingly important role. We decided to focus on these processes in our discussion, also highlighting the most important results.

### Interpretation of results

Transformations of the modern world (interpenetration of cultures, the chaos of values) affect the moral development of adolescents and the specificity of behaviours in which they engage. New theories, formulated in opposition to the classic theories of moral development, even talk about the plasticity of ethical behaviours (Krebs and Denton 2005). According to some authors, globalization brings more opportunities than threats for young people (Arnett 2002). It does limit, however, the range of life choices through the lifestyles imposed by popular culture, especially the ones promoted on the Internet. These dynamic processes refer in particular to sexuality. The research into the sexual morality of adolescents should be situated in this context, while the research into beliefs on sexuality-related norms should take on an empirical dimension.

Research on sexuality-related norms has become more frequent in the last few years, encompassing various cultural circles and age categories of respondents, including adolescents from Eastern and Central Europe (Cislaghi and Heise 2020; Komorowska-Pudło 2013; Pulerwitz et al. 2019). Blocks of questions have been tested mainly on the adult population and relate to sexual violence, acceptance of sexual minorities or people living with HIV, and attitudes towards the sexuality of the elderly or mentally disabled (Izdebski 2020; Perrin et al. 2019). The analysis of scales provides grounds for interpreting complex conceptual constructs and establishing them in a theoretical framework. In this research, we identified three scales of sexual norms: stereotypic gender norms, restrictive sexual norms, and permissive sexual norms. The application of the gender norms scale pertains to one of the essential dimensions of contemporary moral transformations. Moreau et al. (2019a) identified two domains in this area, labelled “Adolescent Romantic Expectations” and “Sexual Double Standards”. The permissive sexual norms scale which we used refers to the behaviours of electronic media users, which narrows down the subject matter (Vangeel et al. 2020). This is, however, an important area outside adult control, where adolescents try to build their own normative system. Modern cultural messages overlap with tradition and upbringing in a certain value system, which in Poland is strongly linked with religious values. It is not by accident, then, that the central place in the constructed popular culture model has been taken by traditional sexual morality (Izdebski 2020; Wąż 2019).

The presented survey was conducted among older adolescents just before, or soon after, sexual initiation. Adolescents who scored higher on the restrictive sexual norms scale were more likely to defer the decision to start sexual activity. Out of the three scales, only permissive sexual norms did not lead to engaging in sex accompanied by feelings of love. Romantic relationships in adolescence have been the subject of many studies (Perrin et al. 2019; Rauer et al. 2013). It has been demonstrated that they can be a source of social support and can lead to better intimate relationships in adulthood (Collins et al. 2009). On the other hand, they can encourage earlier sexual initiation or acceptance of relationship violence (De Graaf et al. 2011; Lehnart et al. 2010). Negative experiences may, in turn, lead to avoiding emotional involvement and commitment. The nationwide studies conducted in Poland for over 20 years now, which take into account various birth cohorts (Izdebski 2020), have shown that the current young generation is less likely to associate sexual initiation with love, which may reflect the progression of cultural change.

Extending the classic analyses of the process of socialization, reference was made to selected factors empowering the individual, such as a sense of the meaning of life, coherence, and self-esteem. In the group of psychosocial factors discussed, peer support, a sense of the meaning of life and self-esteem correlated relatively strongly with moral norms, but not with permissive sexual norms. This is in line with studies which have underscored the significance of peers in social support and in shaping social identity. Peer behaviours affect personal attitudes, while social norms reflect beliefs about what other people do and accept (Bandura 1998; Fishbein and Ajzen 1975; Cislaghi and Heise 2020). As was noted by Collins et al. (2009), the divergence between personal attitudes and norms may lead to actions that are not compliant with personal beliefs. The negative correlation between the feeling of self-esteem and restrictive sexual norms is an interesting result. Adolescence is a period when discrepancies often occur in the perception of moral, religious, and legal norms. A lowered sense of self-esteem may be in mutual relationship with a lack of knowledge, and skills for coping with difficulties (Bandura 1998; Meilstrup et al. 2016).

The key variable identified in the analyses seems to be restrictive sexual norms whose variability is determined by the remaining categories of norms, and other individual and social factors. Comparing the results of logistic regression with the path model, it may be assumed that sexual norms may be mediators of the association between individual or environmental factors and sexual initiation. However, the complex model of determinants of sexual initiation taking into account psychosocial factors and moral norms requires additional analysis.

### Limitations of the study

The above results should be interpreted in the context of a number of limitations. The cross-sectional design does not allow us to make any causal inferences, and the direction of relationships in the path model was pre-assumed. We considered only selected psychosocial determinants, without any consideration of socioeconomic variables. Moreover, attitude towards religion, which has an influence on manifesting more-or-less traditional beliefs, is a missing factor which is worth including in future research. Less attention has been devoted to gender-dependent differences despite a clear accent on this topic. Nevertheless, raising this subject in terms of Polish realities in this cultural sphere, and on the large sample, seems an important research undertaking that needs to be continued.

### Conclusions and implications for further research and practice

Regarding the main research questions, our study confirms that beliefs connected to sexuality are related to the indicators of sexual initiation, especially in the field of restrictive sexual norms. We have also confirmed the mutual dependence between three kinds of norms and their psychosocial determinants. A direct link with self-esteem and an indirect link with the peer environment seem a particularly interesting result. A number of gender-related differences have been reported, which require in-depth analyses. When we started these analyses, we expected to obtain a number of intercorrelated scales. However, we did not think that one of them would take a central position (restrictive norms) and be more strongly linked to psychosocial factors than the others. It was also difficult to anticipate that another scale (permissive norms) would not correlate with these psychosocial factors at all. A diagnosis of sexuality-related social norms should be carried out systematically to take account of the effects of globalization and the occurrence of phenomena which radically alter young people’s attitudes towards sex and sexual partners.

The social norms concerning sexual morality should be included in the analyses of the sexual behaviours of older adolescents, taking into account also complex correlation mechanisms. Such research would have great practical implications. It could provide foundations for developing effective strategies and programmes for sexual education and for the promotion of better sexual and reproductive health.

## Electronic supplementary material

Below is the link to the electronic supplementary material.

Supplementary material 1 (DOCX 16 kb)
