# Supplementary material for: Psychosocial determinants of sexual norms and their impact on sexual debut in Polish adolescents
Source: Int J Public Health. 2020 Sep 2;65(8):1393–401. doi: 10.1007/s00038-020-01470-8 (PMC7588370; doi:10.1007/s00038-020-01470-8)
Supplement: Supplementary file 1 — Supplementary material 1 (DOCX 16 kb) [file 38_2020_1470_MOESM1_ESM.docx]

**International Journal of Public Health**

**PSYCHOSOCIAL DETERMINANTS OF SEXUAL NORMS**

**AND THEIR IMPACT ON SEXUAL DEBUT IN POLISH ADOLESCENTS**

Supplemental Table 1. Final version of developed sexual norms scales (Poland, 2015)

| Name of scale | Items |
| --- | --- |
| *Stereotype gender norms scale (SGN)* | A boy should make the first move to get to know a girl |
|  | Girls like boys who are tender and caring |
|  | A boy should defend a girl if she has been insulted by another boy |
| *Restrictive sexual norms scale (RSN)* | You should avoid sexual contacts with someone you do not love |
|  | I would like to be in one long-term relationship with someone who is “the one” |
|  | There are many ways – not only having sex – to show someone close to us that we care about them |
|  | If a girl/boy wants to wait with sex until marriage, you should respect that |
| *Permissive sexual norms scale (PSN)* | Ending a relationship with an e-mail or a text message is a good solution |
|  | There is nothing wrong with uploading your own „nude” photos or getting undressed in front of a webcam if someone likes it |
|  | Arranging a date with someone you met online can be an interesting event |

*response categories: 1 – strongly disagree; 2-4 not labelled; 5-strongly agree

Supplemental Table 2. Standardized regression estimates (beta) in the path models estimated in the total sample and for boys and girls (Poland,2015)

| Path in the model | | | Total | | Boys | | Girls | |
| --- | --- | --- | --- | --- | --- | --- | --- | --- |
|  |  |  | beta | p | beta | p | beta | p |
| FS | <--- | FC | 0.602 | <0.001 | 0.513 | <0.001 | 0.691 | <0.001 |
| PS | <--- | SOC | 0.086 | 0.010 | 0.139 | 0.002 | 0.093 | 0.058 |
| PS | <--- | PIL | 0.137 | <0.001 | 0.170 | <0.001 | 0.097 | 0.045 |
| PS | <--- | FS | 0.168 | <0.001 | 0.206 | <0.001 | 0.120 | 0.011 |
| SGN | <--- | PSN | -0.339 | <0.001 | -0.292 | <0.001 | -0.282 | <0.001 |
| SGN | <--- | PS | 0.106 | <0.001 | 0.046 | 0.273 | 0.152 | <0.001 |
| SGN | <--- | PIL | 0.061 | 0.040 | 0.122 | 0.003 | 0.035 | 0.424 |
| SE | <--- | SOC | 0.293 | <0.001 | 0.261 | <0.001 | 0.269 | <0.001 |
| SE | <--- | PIL | 0.354 | <0.001 | 0.340 | <0.001 | 0.384 | <0.001 |
| SE | <--- | FS | 0.068 | 0.012 | 0.071 | 0.060 | 0.072 | 0.071 |
| RSN | <--- | SGN | 0.270 | <0.001 | 0.237 | <0.001 | 0.266 | <0.001 |
| RSN | <--- | PSN | -0.333 | <0.001 | -0.293 | <0.001 | -0.294 | <0.001 |
| RSN | <--- | PS | 0.065 | 0.020 | 0.035 | 0.376 | 0.059 | 0.161 |
| RSN | <--- | FS | 0.061 | 0.029 | 0.121 | 0.002 | 0.007 | 0.875 |
| RSN | <--- | SE | -0.130 | <0.001 | -0.095 | 0.015 | -0.087 | 0.039 |
| PIL | <--- | SOC | 0.344 | <0.001 | 0.346 | <0.001 | 0.321 | <0.001 |
| PIL | <--- | FC | 0.183 | <0.001 | 0.169 | <0.001 | 0.197 | <0.001 |
| SOC | <--- | FS | 0.126 | <0.001 | 0.127 | <0.001 | 0.139 | <0.001 |
| SOC | <--- | FC | 0.173 | <0.001 | 0.163 | <0.001 | 0.165 | <0.001 |

Name of scales: SGN – stereotype gender norms; RSN – restrictive sexual norms; PSN – permissive sexual norms; FS – family support; FC – family communication; PC – peer support; SOC – coherence; PIL – purpose in life; SE – self-esteem;
